# Galectins: Their Network and Roles in Infection/Immunity/Tumor Growth Control 2021

**DOI:** 10.3390/biom12091255

**Published:** 2022-09-07

**Authors:** Toshio Hattori

**Affiliations:** Research Institute of Health and Welfare, Kibi International University, Takahashi 716-8508, Japan; hattorit@kiui.ac.jp

Galectins constitute a protein family of soluble and non-glycosylated animal lectins that show a β-galactoside-binding activity via a conserved sequence of approximately 130–140 amino acids located in the carbohydrate recognition domain (CRD). To date, fifteen members, named in the order of their identification, have been identified in mammals. The galectin family members are classified into three types on the basis of their structures. Galectins are stored in the cytoplasm of many types of immune and stromal cells that occur at the entry sites of pathogenic micro-organisms, including fibroblasts, keratinocytes, endothelial cells, and mucosal membrane epithelial cells. Despite the highly conserved nature of galectin CRDs, subtle yet significant differences occur in the binding affinity between different members of the galectin protein family. Galectins are also released from injured cells and express a variety of activities under pathological conditions. This Special Issue aims to present studies that describe novel aspects of galectins.

Kuśnierz-Cabala et al. described “Diagnostic Significance of Serum Galectin-3 in Hospitalized Patients with COVID-19” [1]. This is the second report showing the significantly higher serum galectin-3 (Gal-3) in patients with COVID-19 pneumonia and in those requiring treatment in the ICU as compared to the cases with less severe diseases and to the healthy population. Highly significant positive correlations were shown between serum Gal-3 concentrations and the studied inflammatory markers, including IL-6, PTX-3, and ferritin, and the endothelial dysfunction marker, sFlt-1. Another study showed significantly higher plasma Gal-3 concentrations in 23 COVID-19 patients as compared to 15 healthy controls [2]. Gal-3 is involved in innate immunological reactions to infections, serving as a pattern-recognition receptor, a danger-associated molecular pattern molecule, and an immunomodulator [3].

Plasma levels of Gal-9 are elevated in various disaster-related infectious diseases such as dengue, malaria, and AIDS/TB [4]. Gal-9 is susceptible to cleavage of its linker-peptides by several proteases. It is also interesting that truncated-Gal-9 (Tr-Gal9) levels showed a high discriminating power between the healthy controls, COVID-19 infected and COVID-19 pneumonia patients [5]. A dengue virus infection study also demonstrated Gal-9 as a potential DAMP [6].

Finally, it should be mentioned that both Gal-3 and Gal-9 are potential immune checkpoint molecules, because Gal-3 interacts with LAG3 and the interaction of Gal-9 Tim-3 induces either apoptosis or suppression of T cell effector functions via engagement with its receptor TIM-3. In agreement, Gal-9 knockout mice mount a more robust and vigorous virus-specific immune response, resulting in rapid viral clearance [7].

Wałek et al. wrote “SerumGalectin-3 Concentration Reflects Left Atrial Remodeling and Function in Patients with Persistent Atrial Fibrillation” [8]. Atrial fibrillation (AF) is the most common persistent supraventricular tachyarrhythmia [9]. It is associated with an increased risk of developing heart failure, stroke and premature death [10]. This study included 63 patients scheduled for elective direct current cardioversion (DCCV) due to persistent AF. DCCV was successful in 43 (68.3%) patients and recovery of sinus rhytm was achieved. The concentration of Gal-3 significantly negatively correlates with the size, systolic function, and compliance of the left atrial wall in patients with persistent AF. One of the most important pathways of Gal-3’s activity is its effect on fibroblasts and collagen synthesis. Gal-3 stimulates collagen synthesis and thus contributes to the impairment of the systolic and diastolic functions of the heart muscle [11]. The concentration of Gal-3 negatively correlates with the size and systolic function of the LV in patients with persistent AF. The assessment of Gal-3 concentration in patients with persistent AF may help in the assessment of left atrial remodeling.

Dr. Meggy described the influence of Gal-9 treatment on the phenotype and function of NK-92MI cells. The cytotoxic activity of the natural killer (NK) cells can be triggered without prior sensitization or immunization in a major histocompatibility complex (MHC)-unrestricted manner and regulated by various activating and inhibitory receptors, depending on the presence of their ligands on the target cells and the activation state of the NK cells [12]. For tumor therapy, adoptive transfer of expanded and in vitro-activated NK cells has been widely used [13]. The amplified natural killer cell activity was also applied to adult T cell leukemia (ATL) [14], HTLV-1 infected leukemia, because the NK-cell-inhibitory molecule HLA-Class I gene is hypermethylated and silenced in many ATL cells [15]. They described that the expression of the TIM-3 immune checkpoint receptor can be induced on NK-92MI cells by recombinant Gal-9 treatment, and the elevated level of TIM-3 receptors may mark a dose-dependent activation of these cells, which is strongly masked in the presence of FCS-containing media, but is unmasked when 10% ABS is applied as a culture supplement. The authors suggested to avoid using FCS in their activity examining the biological and regulatory role of Gal-9 or other members of the galectin family.

Campanero-Rhodes et al. examined the binding of four human galectins to the Gram-negative bacteria [16]. Galectins have been shown to bind to the surfaces of some pathogens and products released by the pathogens [17]. These can result in either direct effects on growth of the pathogens or immune responses against them. In this manuscript, binding assays to microarray-printed bacteria revealed that galectins-3, -4, and -8, but not galectin-1, bind to Gram-negative bacteria *Klebsiella pneumoniae* (Kpn) and non-typeable *Haemophilus influenzae* (NTHi) cells, and confocal microscopy attested binding to bacterial cells in suspension. They illustrated galectins’ versatility for recognizing different bacterial structures, and point out the occurrence of so far overlooked galectin ligands on bacterial surfaces. Galectins’ plasticity in targeting diverse ligands in multifarious pathogens, including Gram-negative and -positive bacteria, virus, fungi, and parasites, points to a key role of these lectins in immune protection.

Huang et al. described the immunosuppressive roles of Gal-1 in the tumor microenvironment [18]. Gal-1 is expressed classical Hodgkin’s lymphoma, head and neck cancer, all types of human glioma, pancreatic ductal adenocarcinoma cells, lung cancer, breast cancer and melanoma [18]. In breast cancer, tumor-derived Gal-1 promotes an immunosuppressive breast cancer microenvironment by increasing the frequency of CD4^+^CD25^+^ Foxp3^+^ Treg cells within the tumor, draining lymph nodes, spleen, and lung metastases [19]. The mechanistic studies discussed in this review revealed that Gal-1 contributes to an immunosuppressive tumor microenvironment by inducing apoptosis in effector T cells. Thus, the manipulation of the Gal-1 signaling pathways provides a new avenue for improving checkpoint blockade immunotherapy outcomes.

To conclude, this Special Issue described the novel activities of galectins. Galectins have been present in various organisms since ancient times and maybe the root of their complexity. Further research is desired.
